# The Role of Tactile Stimulation for Expectation, Perceived Treatment Assignment and the Placebo Effect in an Experimental Nausea Paradigm

**DOI:** 10.3389/fnins.2019.01212

**Published:** 2019-11-13

**Authors:** Simone Aichner, Anja Haile, Verena Hoffmann, Elisabeth Olliges, Matthias H. Tschöp, Karin Meissner

**Affiliations:** ^1^Institute of Medical Psychology, Faculty of Medicine, LMU Munich, Munich, Germany; ^2^Division of Health Promotion, Coburg University of Applied Sciences, Coburg, Germany; ^3^Institute for Diabetes and Obesity, Helmholtz Diabetes Center, Helmholtz Zentrum München, Munich, Germany; ^4^German Center for Diabetes Research (DZD), Munich, Germany; ^5^Division of Metabolic Diseases, Department of Medicine, Technische Universität München, Munich, Germany

**Keywords:** placebo effect, expectation, nausea, motion sickness, tactile stimulation, acupuncture

## Abstract

**Introduction:**

Tactile stimulation during a placebo treatment could enhance its credibility and thereby boost positive treatment expectations and the placebo effect. This experimental study aimed to investigate the interplay between tactile stimulation, expectation, and treatment credibility for the placebo effect in nausea.

**Methods:**

Ninety healthy participants were exposed to a 20-min vection stimulus on two separate days and were randomly allocated to one of three groups on the second day after the baseline period: Placebo transcutaneous electrical nerve stimulation (TENS) with tactile stimulation (*n* = 30), placebo TENS without tactile stimulation (*n* = 30), or no intervention (*n* = 30). Placebo TENS was performed for 20 min at a dummy acupuncture point on both forearms. Expected and perceived nausea severity and further symptoms of motion sickness were assessed at baseline and during the evaluation period. At the end of the experiment, participants in the placebo groups guessed whether they had received active or placebo treatment.

**Results:**

Expected nausea decreased significantly more in the placebo groups as compared to the no treatment control group (interaction *day* × *group*, *F* = 6.60, *p* = 0.003, partial η^2^ = 0.20), with equal reductions in the two placebo groups (*p* = 1.0). Reduced expectation went along with a significant placebo effect on nausea (interaction *day* × *group*, *F* = 22.2, *p* < 0.001, partial η^2^ = 0.35) with no difference between the two placebo groups (*p* = 1.0). Twenty-three out of 29 participants in the tactile placebo group (79%) but only 14 out of 30 participants (47%) in the non-tactile placebo group believed that they had received the active intervention (*p* = 0.015). Bang’s blinding index (BI) indicated random guessing in the non-tactile placebo group (BI = 0; 95% CI, −0.35 to 0.35) and non-random guessing in the direction of an “opposite guess” in the tactile placebo group (BI = −0.52; 95% CI, −0.81 to −0.22).

**Conclusion:**

Tactile stimulation during placebo TENS did not further enhance positive treatment expectations and the placebo effect in nausea but increased the credibility of the intervention. Further trials should investigate the interaction between perceived treatment assignment, expectation, and the placebo effect during the course of a trial.

## Introduction

In randomized controlled trials (RCT), the specific effect of acupuncture compared with placebo acupuncture is usually small. An individual patient data meta-analysis with nearly 18.000 patients revealed standardized differences (SD) for the specific effect of acupuncture between 0.15 and 0.23 SD for various chronic pain conditions (Vickers et al., 2012). Compared to no treatment, however, the overall improvement after acupuncture is usually large (Linde et al., 2010a, b; Meissner et al., 2013). The small specific effect of acupuncture compared to the large overall effect indicates the involvement of “non-specific” factors, such as expectation and tactile stimulation, which contribute to the success of acupuncture treatment.

There is increasing evidence that positive treatment expectations influence the non-specific response to acupuncture treatment. In four large RCT of acupuncture, for example, patients with chronic pain conditions and high outcome expectations at baseline showed larger pain reduction at follow-up regardless of whether they were allocated to active acupuncture or placebo acupuncture (Linde et al., 2007). Research from the last decades clearly indicates that the effects of expectation, referred to as “placebo effects,” are deeply rooted in our brains. Placebo effects in pain, for example, are accompanied by the activation of a specific pain modulating network that extends from cortical areas to the spinal cord (Geuter et al., 2017). Expectation can be formed via various mechanisms, including verbal suggestions and conditioning (Colloca and Miller, 2011). Also the non-verbal cues of a treatment, such as prizing, labeling, and dosing, can increase outcome expectations and thus the placebo effect (Meissner and Linde, 2018). Placebo effects have been demonstrated for various clinical conditions including nausea (Quinn and Colagiuri, 2014, 2016; Müller et al., 2016), depression and anxiety (Meyer et al., 2015; Peciña et al., 2015; Rutherford et al., 2016), Parkinson’s disease (De la Fuente-Fernández et al., 2001; Benedetti et al., 2004; Lidstone et al., 2010), as well as for gastrointestinal and cardiovascular systems (Meissner, 2009, 2011; Meissner and Ziep, 2011; Meissner et al., 2011; Ronel et al., 2011; Meissner, 2014; Rief et al., 2017) and immune responses (Hadamitzky et al., 2018).

In addition to positive expectations, skin penetration during needling appears to contribute to the non-specific effects of acupuncture. A re-analysis of the individual patient data meta-analysis by Vickers et al. (2012) revealed a considerably smaller specific effect of acupuncture in RCTs with control groups using skin penetrating needles (SD acupuncture vs. control 0.17, 95% CI 0.11–0.23; *n* = 9) compared to control groups using non-penetrating needles (SD acupuncture vs. control 0.43, 95% CI 0.01–0.85; *n* = 4) (MacPherson et al., 2014). An update of the individual patient data meta-analysis by Vickers et al. (2018) further confirmed this finding, with smaller effects sizes for sham controlled trials that used a penetrating needle in the sham groups compared to trials that used non-penetrating or non-needle sham (difference in SD −0.30, 95% CI −0.60, −0.00, *p* = 0.047). The authors argue that this difference could be either due to the unblinding of patients in the control groups with non-penetrating needles, or to the physiologic effects of skin-penetrating placebo needles, which may have still some therapeutic activity against pain. They argue further that the possibility of unblinding is unlikely, since non-penetrating needles, such as the Streitberger needle, have been confirmed as credible placebos (MacPherson et al., 2014).

However, an imbalance due to differences in blinding could still occur: Patients receiving penetrating needles may be more prone to believe that they have received true acupuncture than patients receiving non-penetrating needles. An “opposite guess” – that is, a higher probability to guess “active treatment” than would be expected by chance (Bang et al., 2010) – could strengthen the outcome expectations of patients in the control groups of RCTs and thereby enhance the placebo effect. First support for this notion comes from experimental placebo studies, showing that “active” placebos that mimic the side effects of pharmacological drugs were more effective in reducing pain than placebos without such side effects (Bjorkedal and Flaten, 2011; Rief and Glombiewski, 2012).

To better understand the role of tactile stimulation, expectation, and perceived treatment assignment for the overall response to acupuncture point stimulation, we conducted a placebo study using an established experimental nausea design (Gianaros et al., 2001; Levine et al., 2006). We recently showed that a placebo transcutaneous electrical nerve stimulation (TENS) stimulation at a dummy point, which elicited slight tactile stimulation, induced a large placebo effect on experimentally induced nausea in comparison to a no-treatment control condition in female participants (Müller et al., 2016). In the present study, we focused on the role of tactile stimulation for the size of the placebo effect and included a second placebo group without tactile stimulation by the TENS device. We hypothesized that tactile stimulation during the placebo intervention would enhance the placebo effect in nausea due to the development of higher outcome expectations. With regard to blinding effectiveness, we expected that the tactile stimulation would lead more participants to believe that they had received the active treatment (“opposite guess”) as compared to the placebo TENS intervention without tactile stimulation, for which we expected a “random guess” (Bang et al., 2010).

## Materials and Methods

### Participants

Healthy adult participants between 18 and 50 years with a history of motion sickness [score ≥80 in Motion Sickness Susceptibility Questionnaire (MSSQ); Golding, 1998] were recruited. Further inclusion criteria were normal or corrected-to-normal vision and hearing, right-handedness, and normal weight (BMI 18–25 kg/m^2^). Exclusion criteria comprised implanted devices (e.g., pacemaker, insulin pump) or metal implants, a history of diseases of the inner ear (e.g., Morbus Menière, acute hearing loss), blood-clotting disorders or a tendency for thromboembolic diseases, and the presence of skin disease, diabetes, cardiovascular disease, epilepsy, or cancer. Further exclusion criteria comprised surgery during the past 4 weeks, current pregnancy or breast feeding, alcohol or drug abuse, inability to imply with the specific instructions, the regular intake of drugs except of hormonal contraceptives, thyroid hormones, and anti-allergic drugs, and anxiety and/or depression scores above the clinically relevant cut-off, as assessed with the Hospital Anxiety and Depression Scale (Zigmond and Snaith, 1983). Participants, who fulfilled all inclusion criteria and none of the exclusion criteria, were invited to participate in a 20-min screening session, during which their susceptibility for the visual vection stimulus was tested. Participants, who developed at least moderate nausea (≥“5” on a 11-point NRS, with “0” indicating “no nausea” and “10” indicating “maximal tolerable nausea”), were invited to participate in the core experiment.

The study protocol was approved by the ethical committee of the Medical Faculty at Ludwig Maximilian University of Munich and was registered retrospectively at the German Clinical Trials Register (no. DRKS00015192). All participants provided written informed consent.

### Study Design

The experiment consisted of a baseline session (day 1) and a testing session (day 2) on two separate days at least 24 h apart. On day 2 after the resting period, participants were randomly allocated to one of four treatment arms: placebo intervention with somatosensory stimulation (*n* = 30), placebo intervention without somatosensory stimulation (*n* = 30), no treatment (*n* = 30), or active treatment (*n* = 10; results not reported). The active treatment arm was implemented to avoid deceptive administration of the placebo treatment. All groups were stratified by sex (50% women, 50% men).

### Experimental Procedure

Participants were tested on two separate days at least 24 h apart (median, 7 days) at the same daytime between 2:00 to 7:00 pm and were instructed to fast at least 3 h before the experiment. Nausea was induced through a standardized visual presentation of alternating black and white stripes with circular motion at 60°, which induces a circular vection sensation (Napadow et al., 2012). The visual stimulus was projected onto a semicylindrical and semitransparent screen placed around the volunteer at a distance of 30 cm to the eyes. Participants were asked to keep their eyes open and look straight ahead without fixating the stripes.

On both testing days upon arrival in the laboratory, participants were seated in a recliner and asked to fill out several questionnaires. Electrodes for psychophysiological assessments were placed, and an indwelling catheter was fixed at the forearm to allow for repeated blood drawings (results of physiological parameters will be reported elsewhere). On day 1, the session started with a 10-min baseline period, followed by a 10-min resting period and a 20-min presentation of the visual vection stimulus. The session ended with a 15 min resting period. On day 2 after the 10-min baseline period, participants were randomized to one of the experimental groups. The experimenter opened the first randomization envelope and delivered standardized information according to group allocation (“treatment” or “no treatment”). Then a medical assistant opened the second randomization envelope, placed the TENS electrodes according to group allocation and started the TENS device for 20 min, if applicable. After 10 min the visual stimulus was presented for 20 min. The experiment ended with a 15-min resting period. For security reasons, the vection stimulus was stopped on both testing days, if nausea ratings indicated severe nausea (ratings of 9 or 10 on 11-point NRS). Table 1 summarizes the time course of the experiment as well as the different times of symptom assessment.

**TABLE 1:** Timeline of the experiment on day 1 and day 2 and time points of behavioral assessments.

		Randomization			
		
	**Baseline**	**Rest 1**	**Nausea 1**	**Nausea 2**	**Rest 2**
					
**Minute**	**1–10**	**11–20**	**21–30**	**31–40**	**41–55**
Visual stimulation (20 min)			ON	ON	
(Placebo) TENS intervention (20 min)		ON^a^	ON^a^		
Perceived nausea (NRS)	X^b^			X^c^	
Perceived dizziness (NRS)	X^b^			X^c^	
SSMS Questionnaire (score)	X^b^			X^c^	
Expected nausea (NRS)		X^d^			
Treatment guess (Verum/Placebo)					X^b^
Perceived treatment efficacy (NRS)					X^b^

*NRS, 11-point numeric rating scale; SSMS, Subjective Symptoms of Motion Sickness. ^*a*^Only day 2, according to group allocation. ^*b*^Last minute. ^*c*^Every minute (ratings were averaged prior to analysis). ^*d*^Immediately after verbal suggestions and applying/testing the TENS device, if applicable.*

### Interventions

Supplementary Table S1 shows the main characteristics of the interventions and the content of the verbal suggestions for each of the three study groups. In short, participants in the treatment groups were informed that the nausea treatment would consist of either an active treatment or a placebo treatment and that the active treatment would reduce nausea by electrical stimulation of an acupuncture point, while the placebo treatment would consist of a placebo acupoint stimulation. Subjects were further informed that the best effects were to be expected when the treatment is begun before exposure to the nauseogenic stimulus (Ezzo et al., 2006). Participants in the no-treatment control group were informed about the rationale and value of a no-treatment control group.

All interventions were conducted using a programmable TENS device (Digital EMS/TENS unit SEM 42, Sanitas, Uttenweiler, Germany). In the placebo groups, two electrodes were attached proximal and distal to a generally accepted dummy point in the context of acupuncture research located on the ulnar side of both forearms (Witt et al., 2012). In the placebo group with somatosensory stimulation (“tactile placebo”), the superficial massage program of the TENS device was turned on for 20 min in order to induce a slight tingling sensation at the electrode site. In the placebo group without somatosensory stimulation (“non-tactile placebo”), electrodes were connected to the TENS device but the device was only allegedly turned on. The active treatment group received real TENS at the acupoint “PC6” (Lee and Fan, 2009) on both forearms for 20 min.

### Randomization and Blinding

Random allocation was accomplished using sealed and numbered envelopes. A person not directly involved in the experiments prepared the randomization envelopes based on a computer-derived randomization list. The interventions were performed in a single-blind design. Participants in the no-treatment control group were necessarily unblinded.

### Ratings and Questionnaires

Expected nausea intensity was rated on 11-point NRS, with “0” indicating “no expected nausea” and “10” indicating “maximal tolerable expected nausea.” Perceived nausea and dizziness intensities were rated at baseline and every minute during the nausea period on 11-point NRSs, with “0” indicating “no nausea/dizziness” and “10” indicating “maximal tolerable nausea/dizziness.” Symptoms of motion sickness were assessed using the “Subjective Symptoms of Motion Sickness” (SSMS) questionnaire (adapted from Graybiel et al., 1968), with scores of 0–3 assigned to responses of none, slight, moderate, and severe for symptoms of dizziness, headache, nausea/urge to vomit, tiredness, sweating, and stomach awareness, respectively. At the end of day 2, participants in the treatment groups were asked to guess whether they had received active or placebo treatment as well as to rate the perceived effectiveness of treatment on an 11-point NRS, with “0” indicating “not effective at all” and “10” indicating “highly effective.” They were furthermore asked to rate on 11-point NRS, how sure they were about their treatment guesses, with “0” indicating “not sure at all” and “10” indicating “very sure.” Bang’s blinding index (BI) was used to estimate the proportion of participants who guess their treatment incorrectly beyond chance level. Random guess exists if the BI’s confidence interval covers 0 (Bang et al., 2010).

### Statistical Analyses

Sample size calculation was performed for baseline-adjusted nausea scores. Assuming a medium effect size partial eta-squared of 0.13 for the difference in baseline-adjusted nausea scores between the tactile and the non-tactile placebo group, 28 subjects per group would be needed to give 80% power to detect a significant difference (with a type 1 error of 5%) (calculated by G^∗^Power Version 3.1.7). We increased the sample size to 30 per group to compensate for possible attrition rates. Statistical analyses were performed with SPSS statistics software (version 24, IBM).

Prior to the analyses, nausea and dizziness ratings were averaged for the evaluation period, which comprised minutes 11 to 20 of visual nausea induction. A period without placebo-TENS intervention was chosen in order to avoid distraction effects by tactile stimulation. Expected nausea as well as nausea, dizziness, and the SSMS sum score were subjected to separate 2 × 3 × 2 mixed-design analyses of variance (ANOVA), with day (day 1: baseline, day 2: intervention) as the within-subject factor and group (placebo TENS with tactile stimulation, placebo TENS without tactile stimulation, no treatment) and sex (male, female) as between-subject factors. Significant effects were followed-up by Bonferroni-corrected *post hoc* tests. For all statistical tests, a *p*-value of ≤0.05 (two-tailed) was considered statistically significant.

## Results

### Sample

**FIGURE 1:**
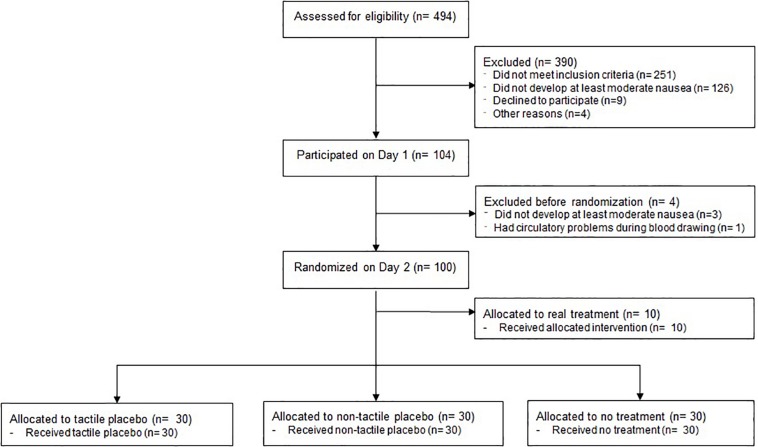
Flow chart.

The flow of study participants is shown in Figure 1. In total, 494 volunteers were assessed for eligibility and 245 were invited to participate in the screening session to assess their susceptibility for the visual vection stimulus. 104 volunteers were included in the study, four of whom were excluded before randomization on Day 2. Hundred participants were randomized and completed the experiment. Analyses were based on the data from 90 participants assigned to placebo treatment or no treatment [45 males, 45 females; age (*M* ± *SD*), 23.5 ± 3.2 years]. The experimental groups were comparable with regard to sociodemographic, physical and psychological characteristics (Table 2).

**TABLE 2:** Sample characteristics at baseline.

	**No treatment (*n* = 30)**	**Non-tactile placebo (*n* = 30)**	**Tactile placebo (*n* = 30)**	***p*^∗^**
Sex, *m/f*	15/15	15/15	15/15	1
Age, mean (*SD*)	23.5 (2.7)	23.8 (3.8)	23.1 (3.0)	0.69
Education (≥high school degree), *n* (%)	27 (90)	30 (100)	29 (97)	0.59
Non-smoker, *n* (%)	26	27	26	0.90
Body mass index	22.3 (2.7)	21.9 (2.2)	21.2 (2.0)	0.31
MSSQ, mean (*SD*)	130.3 (38.4)	139.8 (42.3)	134.5 (37.9)	0.65
HADS-anxiety, mean (*SD*)	4.0 (2.6)	3.8 (2.2)	4.2 (2.2)	0.75
HADS-depression, mean (*SD*)	1.4 (1.6)	1.5 (1.8)	2.0 (1.5)	0.10
STAI-trait anxiety, mean (*SD*)	38.8 (6.4)	38.1 (6.7)	37.4 (6.3)	0.61
PSQ-Stress, mean (*SD*)	31.1 (14.3)	30.9 (19.4)	28.7 (12.1)	0.81

*MSSQ, Motion Sickness Susceptibility Questionnaire; HADS, Hospital Anxiety and Depression Scale; STAI, State-Trait Anxiety Inventory; PSQ, Perceived Stress Questionnaire. ^∗^Results of χ^2^ tests, Kruskal–Wallis tests, or univariate ANOVA, as appropriate.*

### Nausea Expectation

The 2 × 3 × 2 mixed-design ANOVA indicated differential changes of expected nausea from day 1 to day 2 in the experimental groups (interaction *day* × *group*, *F* = 6.60, *p* = 0.003, partial η^2^ = 0.20) without a difference between male and female participants (interaction *day* × *group* × *sex*, *F* = 1.8, *p* = 0.564, partial η^2^ = 0.02). *Post hoc* tests indicated significantly lower levels of expected nausea in both placebo groups in comparison to the no treatment group (Bonferroni-corrected *p*-values 0.002 and 0.021 vs. no treatment for the tactile and the non-tactile placebo groups, respectively). The decrease in expected nausea did not differ between the tactile and the non-tactile placebo groups (*p* = 1.0) (Table 3).

**TABLE 3:** Expected and perceived symptoms (day 1, day 2, and changes) in each experimental group.

	**No treatment (*n* = 30)**	**Non-tactile placebo (*n* = 30)**	**Tactile placebo (*n* = 30)**
**Nausea expectation (NRS 0–10)**			
Control day, mean (*SD*)	6.8 (2.2)	7.6 (1.3)	7.6 (1.0)
Intervention day, mean (*SD*)	7.0 (1.1)	5.6 (1.9)	5.2 (2.4)
Mean change (95% CI)	0.3 (−1.0; 1.6)	−2.1 (−3.3; −1.0)^∗^	−2.8 (−4.0; −1.5)^∗∗^
**Baseline-adjusted nausea score (NRS 0–10)**			
Control day, mean (*SD*)	5.6 (1.5)	6.0 (1.6)	5.2 (2.0)
Intervention day, mean (*SD*)	4.8 (1.8)	2.7 (2.1)	2.0 (2.0)
Mean change (95% CI)	−0.8 (−1.4; −0.2)	−3.2 (−3.9; −2.6)^∗∗∗^	−3.2 (−3.8; −2.6)^∗∗∗^
**Baseline-adjusted dizziness score (NRS 0–10)**			
Control day, mean (*SD*)	5.8 (1.8)	5.7 (1.9)	4.9 (1.8)
Intervention day, mean (*SD*)	5.1 (2.2)	3.1 (2.2)	2.4 (1.7)
Mean change (95% CI)	−0.7 (−1.2; −0.1)	−2.6 (−3.1; −1.9)^∗∗∗^	−2.5 (−3.1; −1.9)^∗∗∗^
**Baseline-adjusted SSMS score (0–18)**			
Control day, mean (*SD*)	6.9 (2.7)	6.6 (3.4)	5.8 (2.6)
Intervention day, mean (*SD*)	6.4 (2.6)	4.1 (2.2)	3.3 (2.2)
Mean change (95% CI)	−0.5 (−1.4; 0.4)	−2.5 (−3.7; −1.4)^∗^	−2.5 (−3.5; −1.51)^∗^

*NRS, numeric rating scale (0–10), average of 11 individual NRS ratings assessed during the target period on the control and intervention days; SSMS, Subjective Symptoms of Motion Sickness Questionnaire. ^∗^*p* < 0.05 (vs. no treatment, Bonferroni-corrected). ^∗∗^*p* < 0.01 (vs. no treatment, Bonferroni-corrected). ^∗∗∗^*p* < 0.001 (vs. no treatment, Bonferroni-corrected).*

### Nausea and Dizziness

**FIGURE 2:**
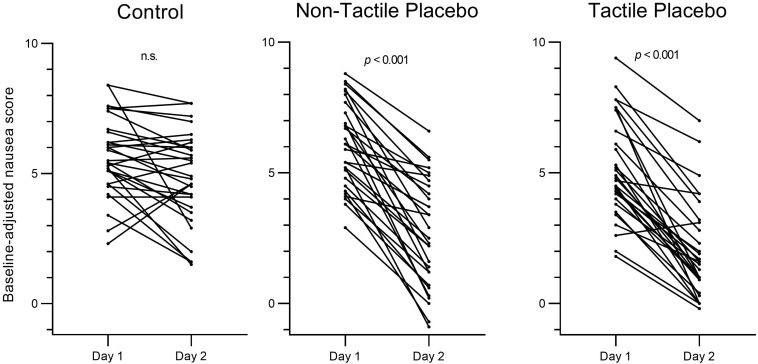
Individual changes in baseline-adjusted nausea score from day 1 (control) to day 2 (intervention) in the control group, the non-tactile placebo group, and the tactile placebo group. Nausea was assessed on 11-point numeric rating scales.

The 2 × 3 × 2 mixed-design ANOVA for baseline-adjusted nausea scores indicated a significant group-by-day interaction (*F* = 22.2, *p* < 0.001, partial η^2^ = 0.35) with no evident sex difference (interaction *day* × *group* × *sex*, *F* = 1.0, *p* = 0.390, partial η^2^ = 0.02). The decrease in baseline-adjusted nausea scores from day 1 to day 2 was significantly larger in the placebo groups as compared to the no treatment group, confirming the occurrence of a placebo effect in nausea (both Bonferroni-corrected *p*’s < 0.001). The placebo effect did not differ between the two placebo groups (*p* = 1.0) (Table 3; Figure 2).

The 2 × 3 × 2 mixed-model ANOVA for baseline-adjusted dizziness scores likewise revealed a significant group-by-day interaction (*F* = 14.2, *p* < 0.001, partial η^2^ = 0.25), without a difference between male and female participants (interaction *day* × *group* × *sex*, *F* = 1.7, *p* = 0.190, partial η^2^ = 0.04). Significantly larger decreases of baseline-adjusted dizziness scores were observed in both placebo groups in comparison to the no treatment group (both Bonferroni-corrected *p*’s < 0.001), while no differences between the two placebo groups occurred (*p* = 1.0) (Table 3).

### Subjective Symptoms of Motion Sickness

The 2 × 3 × 2 mixed-design ANOVA for sum scores in the baseline-adjusted SSMS scores revealed a significant group-by-day interaction (*F* = 7.9, *p* = 0.001, partial η^2^ = 0.16), comparable in size for male and female participants (interaction *day* × *group* × *sex*, *F* = 1.1, *p* = 0.333, partial η^2^ = 0.03). Bonferroni-corrected *post hoc t*-tests indicated significantly larger decreases of baseline-adjusted SSMS scores in both placebo groups in comparison to the untreated control group (*p* = 0.012 and 0.014 vs. untreated controls for the tactile and non-tactile placebo groups, respectively). Again, changes in baseline-adjusted SSMS scores did not differ between the two placebo groups (*p* = 1.0) (Table 3).

### Perceived Treatment Effectiveness and Treatment Guesses in the Placebo Groups

Twenty-three out of 29 participants in the tactile placebo group (79%) believed that they had received the active intervention as compared to 14 out of 30 participants (47%) in the non-tactile placebo group, the difference was significant (χ*^2^* = 6.72, *p* = 0.015). Participants in the two placebo groups were equally sure about their treatment guesses (non-tactile placebo, 5.3 ± 2.6 (*mean* ± *SD*); tactile placebo: 5.9 ± 2.4; *Z* = −1.12, *p* = 0.363). Explorative analyses revealed no relationship between changes in baseline-adjusted nausea scores from day 1 to day 2 and treatment guess (guess “placebo,” −3.0 ± 1.9; guess “verum,” −3.5 ± 1.5; *F* = 1.0, *p* = 0.313).

At the end of the experiment, participants in the tactile placebo group rated the treatment as significantly more effective than did participants in the non-tactile placebo group (*mean* ± *SD*, 6.9 ± 2.2 vs. 5.7 ± 2.5; *Z* = −1.99, *p* = 0.046). At the same time, larger reductions in baseline-adjusted nausea scores from day 1 to day 2 correlated with higher effectiveness ratings in both placebo groups (non-tactile placebo, *r*_*s*_ = −0.446, *p* = 0.013; tactile placebo, *r*_*s*_ = −0.402, *p* = 0.027). An exploratory regression analysis on perceived treatment effectiveness with the factors “type of placebo” and “reduction in baseline-adjusted nausea” included as independent variables revealed that both factors contributed significantly to subjective effectiveness (*F* = 7.2, *p* = 0.001; type of placebo, β = 0.257, *p* = 0.034; reduction in baseline-adjusted nausea, β = −0.377, *p* = 0.002). These results suggest that ratings of perceived effectiveness are driven by both, perceived improvement and sensory characteristics of the placebo intervention.

### Bang’s Blinding Index in the Two Placebo Groups

Bang’s BI in the non-tactile placebo group was 0 (95% CI, −0.35 to 0.35), indicating random guessing (Table 4). In the tactile placebo group Bang’s BI was −0.52 (95% CI, −0.81 to −0.22), indicating non-random guessing in the direction of an “opposite guess,” that is, the probability to guess “active treatment” was significantly higher than would be expected by chance.

**TABLE 4:** Bang’s blinding index for the non-tactile and tactile placebo groups.

	**Guess, *n***			
	
**Assignment**	**Active treatment**	**Placebo treatment**	**Don’t know^∗^**	**Total**
Non-tactile placebo	14 (47%)	14 (47%)	2 (7%)	30
Tactile placebo	21 (72%)	6 (21%)	2 (7%)	29
Total	35	20	4	59

*^∗^Two participants in each placebo group indicated that they were “not sure at all” (NRS = “0”) about their treatment guess and were re-assigned to category “don’t know.”*

## Discussion

In this randomized controlled placebo study, we aimed to vary the credibility of two placebo interventions by combining, or not combining it with tactile stimulation elicited by a TENS device. We hypothesized that 20 min of tactile stimulation would increase positive outcome expectations and thus the placebo effect. Results confirmed a large effect of the tactile stimulation by TENS on the credibility of the placebo treatment: Significantly more participants in the tactile placebo group believed that they had received the active intervention as compared to the non-tactile placebo group. In addition, the tactile placebo intervention was perceived as more effective. Neither expectations nor the placebo effect, however, differed between the two placebo groups.

The placebo effect as the difference between the placebo groups and the no-treatment control group was consistent for different outcome parameters and effect sizes were generally large (partial η^2^, 0.16–0.35; Richardson, 2011). Results thus confirm the findings of our pilot study that placebo TENS induces a large placebo effect in experimentally induced nausea (Müller et al., 2016) and further extend them to male volunteers and to a placebo TENS intervention without tactile stimulation. The medical environment, in which the experiment took place – with many factors present that are known to boost placebo effects, such as a room full of sophisticated electrical equipment as well as prolonged interaction with the experimenters (Burke et al., 2019) – may have contributed to this large placebo effect.

As hypothesized, somatosensory stimulation during the placebo intervention increased blinding effectiveness: Bang’s BI indicated random guessing in the non-tactile placebo group but non-random guessing in the direction of an opposite guess in the tactile placebo group. Our results thus lend support to the view that somatosensory stimulation during acupuncture point stimulation challenges the goal of patient blinding by enhancing the chance for a non-random guess. Given that most placebo acupuncture procedures are associated with random-guesses (Zhang et al., 2015), this discrepancy could result in a problematic blinding scenario with enhanced expectations and placebo effects in the true acupuncture groups (Bang et al., 2010; Chae et al., 2018). Contrary to our expectations, however, tactile stimulation by the TENS device during the placebo intervention did neither enhance outcome expectations nor the placebo effect during the evaluation period. Possibly, participants with opposite treatment guesses after the first placebo application may develop higher treatment expectations only with respect to subsequent placebo interventions. In a recent RCT in depression, for example, perceived treatment assignment affected symptom improvement only in the second half of the trial (Laferton et al., 2018). Furthermore, a large RCT in patients with chronic arm pain found no evidence that sham acupuncture was associated with an enhanced placebo effect during the 2 week placebo run-in period; however, sham acupuncture was significantly more effective than placebo pills during the further 6 weeks of the trial (Kaptchuk et al., 2006). Future studies are warranted to disentangle the putative interaction between expectation, perceived treatment assignment, and the placebo effect during the course of a trial.

Several possible limitations have to be considered. The medical setting of our experiment may have resulted in a ceiling effect, thereby preventing further enhancement of the placebo effect by tactile stimulation. Most acupuncture trials, however, are performed in comparable medical settings, emphasizing the external validity of our results. Furthermore, the gentle touch when placing the electrodes of the TENS device at the participants’ skin could have initiated physiological responses by activating unmyelinated C tactile fibers in the body, resulting in feelings of calm and well-being as well as lower heart rate and blood pressure (Campbell, 2006; Kang et al., 2011; Chae et al., 2018). Such physiological effects may have contributed to the improvement in the placebo groups independently from expectation. However, also the participants in the untreated control group received a variety of skin electrodes to measure the EEG, the EKG and the electrogastrogram and were provided with an indwelling catheter for repeated blood drawings during the experiment. Therefore, the gentle touch when placing the TENS electrodes was not unique to the placebo groups and the only difference between placebo and no treatment groups was the therapeutic meaning of placing the TENS electrodes. Finally, placing the TENS electrodes in the non-tactile placebo group also involved some amount of tactile stimulation and may thereby have enhanced the placebo effect. Compared with 20 min of somatosensory stimulation stimulation in the tactile TENS placebo group, however, somatosensory stimulation in the non-tactile TENS placebo group was considered to be only minor. The differential pattern of treatment guesses in the two placebo groups further supports the conceptual difference between the two placebo interventions.

## Conclusion

Electrical stimulation during a placebo TENS intervention did not enhance the placebo effect in nausea but increased the credibility of the treatment. Further experimental trials are needed to investigate the putative interaction between perceived treatment assignment, expectation, and the placebo effect during the course of a trial.

## Data Availability Statement

The datasets generated for this study are available on request to the corresponding author.

## Ethics Statement

All subjects gave written informed consent in accordance with the Declaration of Helsinki. The protocol was approved by the Ethics Committee of the Medical Faculty (LMU Munich) and was registered at the German Clinical Trials Register (no. DRKS00015192).

## Author Contributions

KM and MT designed the study. SA, AH, and VH conducted the experiment. KM, SA, and EO analyzed and interpreted the data. KM and SA drafted the manuscript. All authors revised the manuscript for critical intellectual content and approved the final version.

## Conflict of Interest

The authors declare that the research was conducted in the absence of any commercial or financial relationships that could be construed as a potential conflict of interest.
