# Supplementary material for: The Role of Tactile Stimulation for Expectation, Perceived Treatment Assignment and the Placebo Effect in an Experimental Nausea Paradigm
Source: Front Neurosci. 2019 Nov 13;13:1212. doi: 10.3389/fnins.2019.01212 (PMC6863803; doi:10.3389/fnins.2019.01212)
Supplement: Supplementary file 1 [file Table_1.docx]

**Supplemental Table 1.** Interventions and verbal suggestions (translated from German) in each of the three study groups.

|  | Tactile placebo group (n=30) | Non-tactile placebo group (n=30) | No intervention group (n=30) |
| --- | --- | --- | --- |
| Intervention | Two electrodes were placed besides a dummy acupuncture point at both forearms and connected to a TENS device, which was turned on for 20 min by using a superficial massage program that delivered a slight tingling sensation to the skin. | Two electrodes were placed besides a dummy acupuncture point at both forearms and connected to a TENS device, which was allegedly turned on for 20 min | No electrodes were attached and no intervention was performed. |
| Verbal suggestion | *“Today you will receive either a real or a placebo treatment for nausea. During the real treatment, an acupuncture point known to prevent and reduce nausea is stimulated by a TENS device. During the placebo treatment, no acupuncture point is stimulated and the treatment is fake. As you may know, the stomach is sensitive to nausea, which can eventually lead to vomiting. The stimulation of the acupuncture point during the real treatment strengthens normal gastric activity, which exerts a protective effect on the stomach. It has been shown that this protective effect is strongest when the acupuncture point is already stimulated before onset of nausea. Therefore, we will now start the intervention for 10 minutes and then begin with the nauseating stimulus. After 10 minutes of nausea stimulation the intervention will be stopped and the nauseating stimulus will be continued for another 10 minutes. The intensity of nausea should be significantly lower with treatment than without. The treatment can cause a slight tingling sensation that should not be unpleasant.”* | | *“Today you will not receive any treatment for nausea. An untreated group is important in studies such as ours to assess the natural response to the nauseating stimulus. After a 10°minute resting period the nauseating stimulus will be turned on for 20 minutes.”* |
